# NORMOLIPEMIC TUBEROUS XANTHOMAS

**DOI:** 10.4103/0019-5154.53190

**Published:** 2009

**Authors:** Ajay Pal Singh, Shilpi Sikarwar, O P Jatav, Khozema Saify

**Affiliations:** *From the Department of Medicine, G.R. Medical College, Gwalior - 474 009, MP*

**Keywords:** *Cutaneous*, *normolipemic*, *tuberous xanthoma*

## Abstract

Xanthomas are often a manifestation of underlying lipid abnormalities. A 50-year-old male presented to our hospital with the lesions of multiple tuberous xanthomas all over the body. Routine investigations and systemic examination were normal. Lipid profile was within normal range and serum protein electrophoresis showed normal pattern. Histopathology from a nodular lesion showed collection of foamy macrophages in the dermis. We present a case of normolipemic tuberous xanthomas, which is an uncommon occurrence.

## Introduction

Xanthomas are tumor-like collections of foamy histiocytes within the dermis. They may be associated with familial or acquired disorders resulting in hyperlipidemia, with lyphoproliferative malignant neoplasms, or with no underlying disorder.[1] Tuberous xanthomas occur as yellow nodules and are frequently associated with hypertriglyceridemia, but they are also seen in patients with hypercholesterolemia (type II).[2]

We present a case of multiple tuberous xanthomas in a subject with normal lipid metabolism and with no associated systemic disorders, which is an uncommon occurrence. This case is being reported because of its rare occurrence. The aim of this report is to emphasize the importance of considering this disease entity in a patient with normal lipid profile.

## Case Report

A 50-year-old male presented in outpatient department with history of yellowish nodular lesions on the trunk and limbs for the past two years. The lesions first appeared as small, yellowish nodules, which were well circumscribed on extensor surface of forearms and arms. After two months, nodular lesions appeared on the back of neck, trunk, thighs, and legs. There was no history of diabetes mellitus, hypothyroidism, or any other systemic disease. The patient was a bidi smoker for the past 30 years. There was no history of similar lesions observed in his parents, siblings, or children.

**Figure 1:**
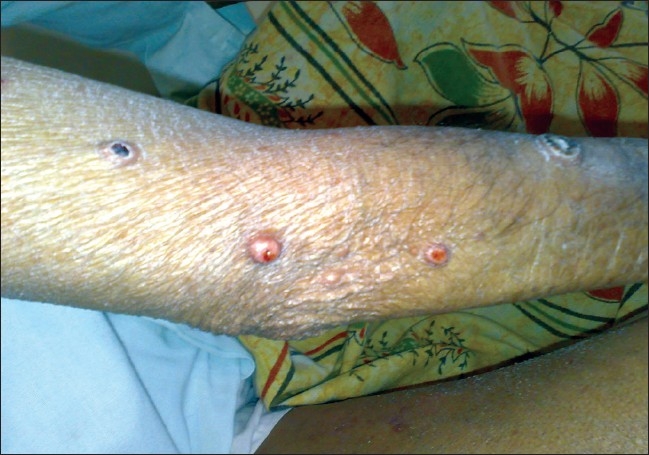
Tuberous xanthomas with central crusting on extensor surface of forearm and arm

**Figure 2:**
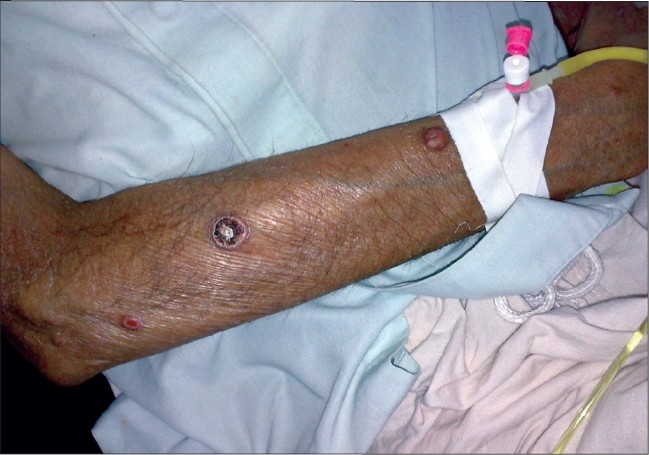
Tuberous xanthomas on extensor surface of arm

**Figure 3:**
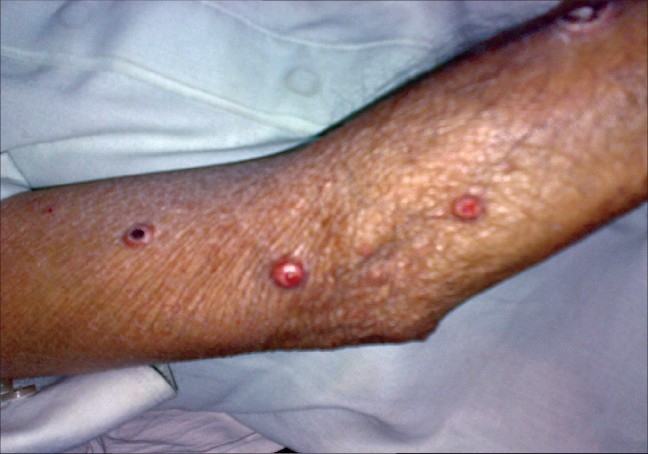
Tuberous xanthomas on extensor surface of left forearm and arm with central crusting

**Figure 4:**
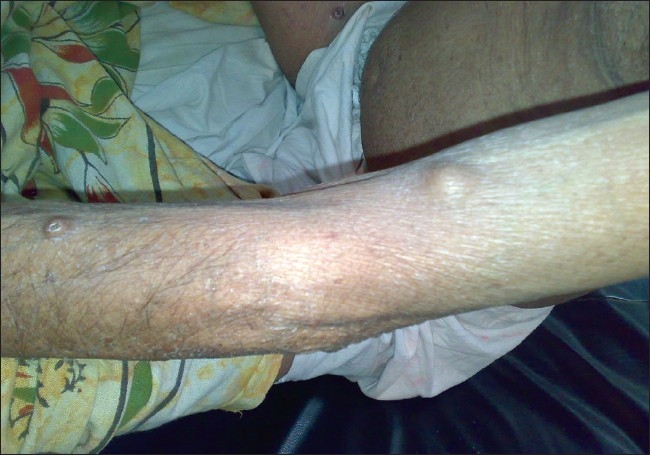
Tuberous xanthomas on left forearm and arm

**Figure 5:**
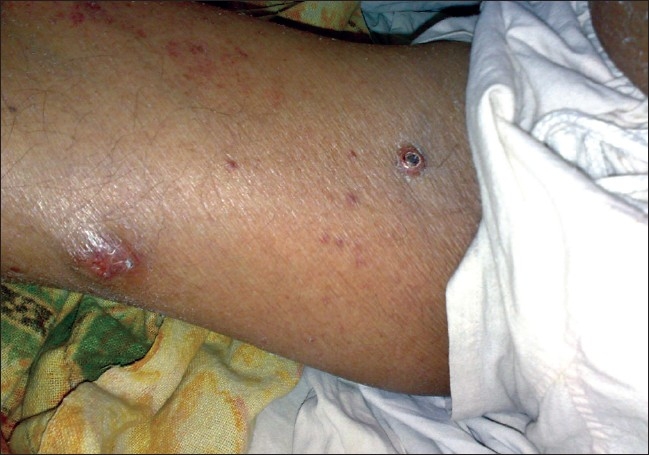
Tuberous xanthomas on flexor surface of right thigh

**Figure 6:**
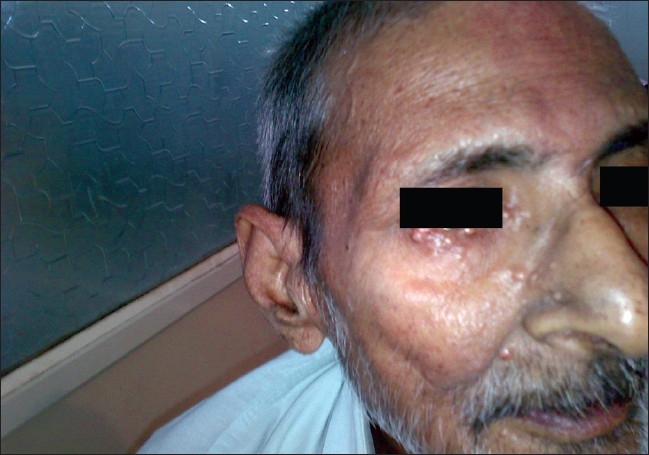
Tuberous xanthomas on face

**Figure 7:**
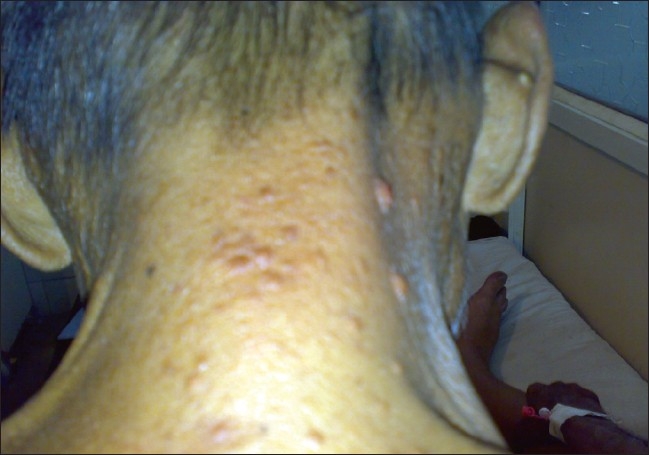
Tuberous xanthomas on neck

**Figure 8:**
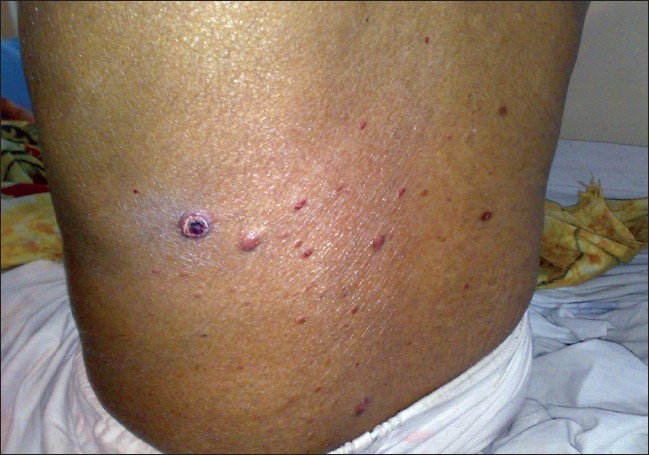
Tuberous xanthomas on back

On examination, the patient looked healthy and had no pallor. Cutaneous examination revealed multiple, firm, nontender, mobile, red-yellow nodules, 1–2 cm in size on the dorsal aspects of forearms and arms, medial aspect of thighs, extensor surface of legs, back of neck, face, and lower back [Figures 1–8]. Some of the lesions were found on forearms, arms, and back had central crusting. Rest of the physical examination was within normal limit except for skin lesions.

**Figure 9:**
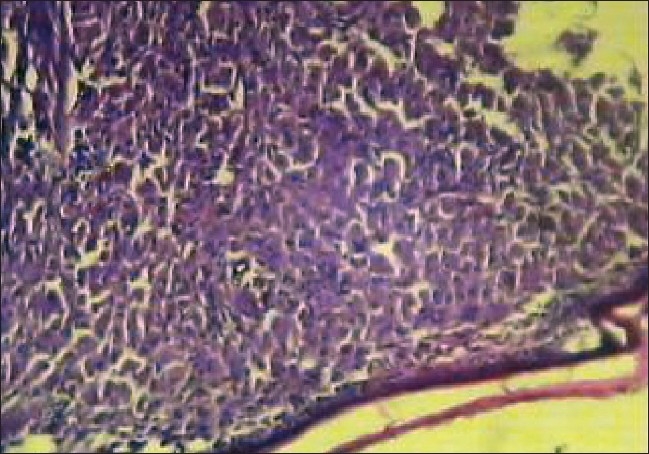
Microphotograph illustrates thinned out epidermis with foamy macrophages and fibrous band [H&E, ×10]

**Figure 10:**
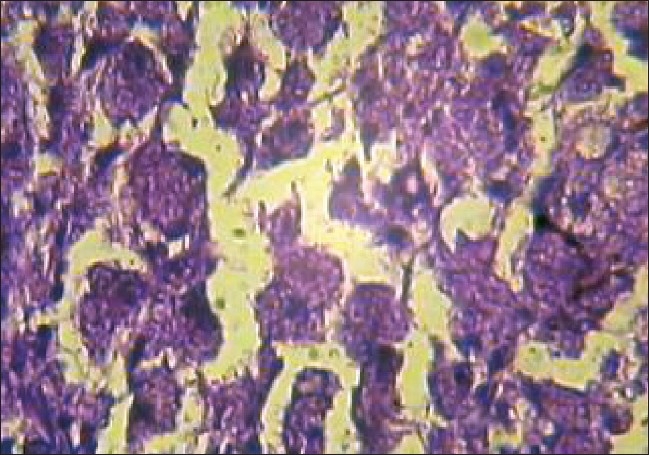
Microphotograph illustrates foamy macrophages [H&E, ×40]

Routine investigations including complete hemogram and urine analysis were normal. Other investigations revealed normal lipid profile, thyroid, liver and kidney function tests, and blood sugar levels. The results of chest X-ray, E.C.G, and ultrasonography of abdomen were normal. Serum protein electrophoresis showed normal pattern. The histopathological evaluation from one of the nodules showed thinned out epidermis with collection of lobules of foamy macrophages separated by fibrous bands [Figures 9 and 10]. The patient was kept on follow up and repeated lipid profile after one month showed almost similar values. Cryotherapy with nitrous oxide was done for large tuberous xanthomas along with oral antioxidants. Patient is responding well to the treatment. The skin lesions are healing and he is still under follow-up.

## Discussion

Xanthomas are tumors or infiltrates of the skin varying from yellow to brown-red colour, which are due to lipid-containing cells in the dermis. Xanthomas may be the symptoms of a general metabolic disease, a generalized histiocytosis, or a local fat phagocytosing storage process. Xanthoma disseminatum and verruciform xanthoma are particular forms of xanthomas that occur in normolipemic patients.[3] Tuberous xanthomas are firm painless, red–yellow nodules. The lesions can coalesce to form multilobated tumors. Tuberous xanthomas usually develop in pressure areas, such as the extensor surfaces of the knees, the elbows, and the buttocks. In our case too, xanthomas are more common in these areas. Tuberous xanthomas are particularly associated with hypercholesterolemia and increased level of LDL.[4] They can be associated with familial dysbetalipoproteinemia and familial hypercholesterolemia type (Frederickson IIa and III hyperlipoproteinemias), and they may be present in some of the secondary hyperlipidemias. However, our patient did not show any type of hyperlipidemia.

Histopathologically, xanthomas are characterized by the presence of vacuolated macrophages in dermis. These macrophages are filled with lipid droplets, which are dissolved and removed from tissue during histologic processing. Tuberous xanthomas can contain prominent fibrosis and occasional cholesterol clefts. Fibrosis is also seen in histopathological examination of a nodule in this patient.

Multiple tuberous xanthomas are characteristically associated with hyperlipidemic states. However, normolipemic xanthomatosis have been reported in the literature, but this entity is uncommon.[3,5–7] Diffuse normolipemic xanthomatosis have normal lipid levels, but are often associated with serious hepatic disease or hematological dyscrasias, especially multiple myeloma.[8] Hu, *et al.* reported unusual normolipemic cutaneous xanthomatosis with IgG gammopathy, hypernephroma, an unusual family cluster of leukemia.[9] Vail *et al*. reported a case of chronic myelomonocytic leukemia with cutaneous xanthomas.[10]

Thus, normolipemic xanthomatosis has been found to be associated with either a systemic disease or malignancy. Our case showed multiple tuberous xanthomas but without any lipid disorder or associated systemic disease or malignancy. Normolipemic tuberous xanthomas has been reported previously also, but its occurrence is very rare.[5,6]

This report emphasizes this disease entity in a patient with normal lipid profile and with no associated systemic disorders.
